# Supplementary material for: Identification of ROH Islands Conserved through Generations in Pigs Belonging to the Nero Lucano Breed
Source: Genes (Basel). 2023 Jul 23;14(7):1503. doi: 10.3390/genes14071503 (PMC10378754; doi:10.3390/genes14071503)
Supplement: Supplementary file 1 [file genes-14-01503-s001.zip › genes-2507530-supplementary.pdf]

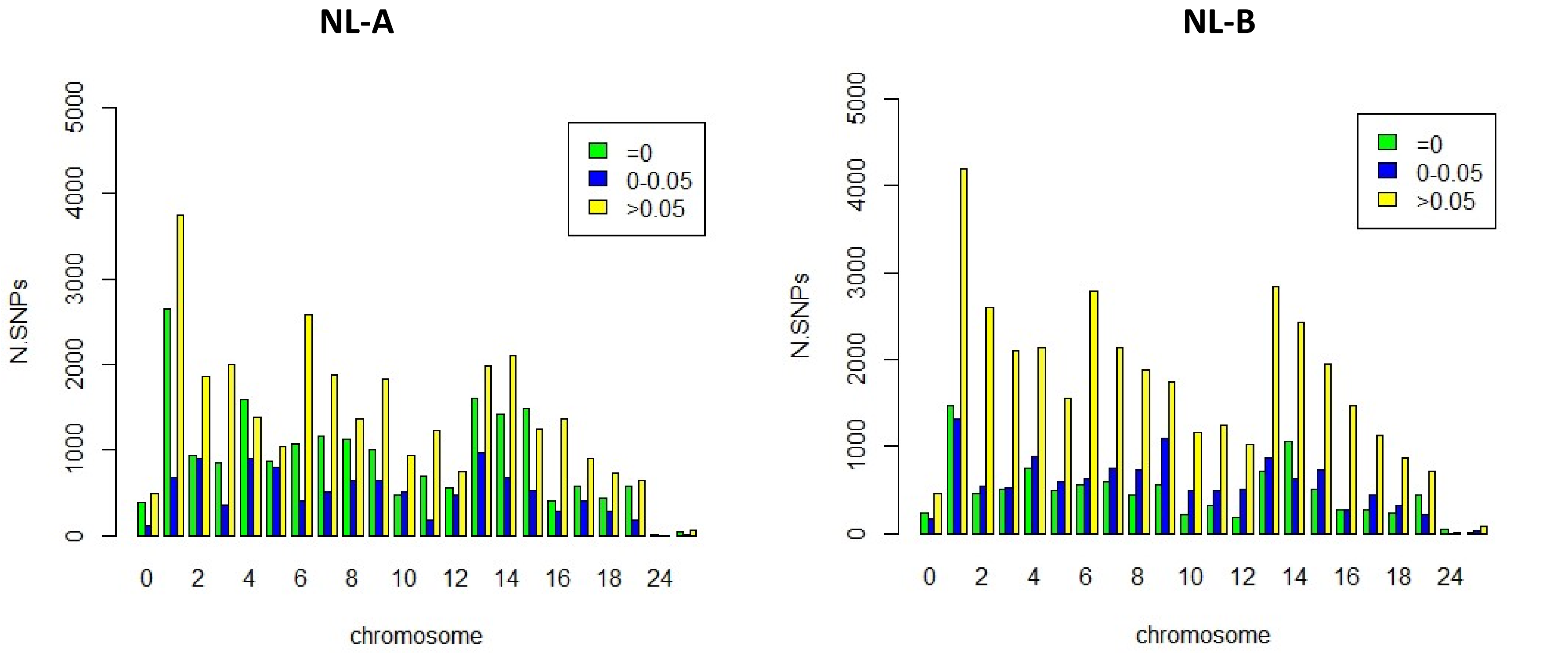

Figure S1. Chromosomal distribution according to the Minimum Allele Frequencies (MAF) of the SNPs in the two groups, NL-A and NL-B, of Nero Lucano pigs (0= non-defined chromosome position, 23=X chromosome, 24= Y chromosome, 25= XY ψ-autosomal region).

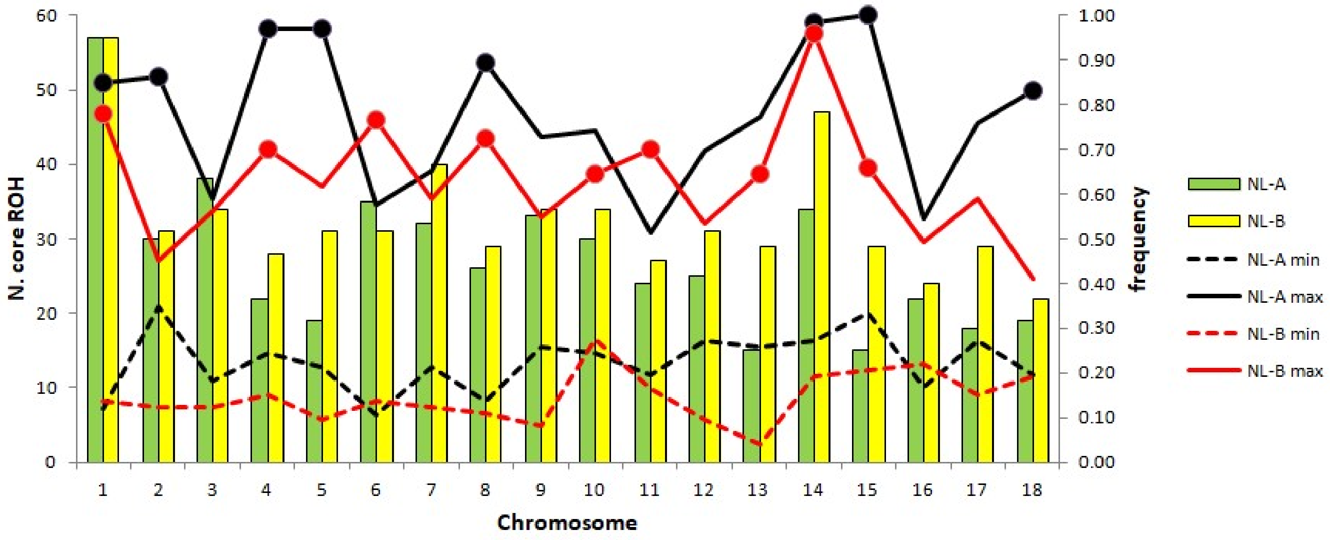

Figure S2. Chromosomal distribution of core ROH (bars), and minimum and maximum frequencies of core ROH (lines) in the two groups, NL-A and NL-B, of Nero Lucano pigs. Dots indicate ROH islands.

Table S1. Chromosomal distribution of the number of core ROH and ROH islands in the two groups, NL-A and NL-B, of Nero Lucano pigs.

SSC	NL-A		NL-B	
	Core ROH	ROHisl	Core ROH	ROHisl
1	57	1	57	1
2	30	1	31	-
3	38	-	34	-
4	22	6	28	6
5	19	3	31	-
6	35	-	31	1
7	32	-	40	-
8	26	1	29	4
9	33	-	34	-
10	30	-	34	2
11	24	-	27	2
12	25	-	31	-
13	15	-	29	1
14	34	2	47	4
15	15	3	29	3
16	22	-	24	-
17	18	-	29	-
18	19	2	22	-
Tot.	494	19	587	24

Table S2. Features of the ROH islands in the two groups, NL-A and NL-B, of Nero Lucano pigs (total overlaps are highlighted in yellow, partial overlaps are in red).

NL-A				
**SSC**	**start**	**end**	**MB**	**NSNP**
1	61286411	63123772	1.84	52
2	138165413	140762514	2.60	97
4	96094167	96682751	0.59	19
	103876353	108603753	4.73	163
	117536160	117687859	0.15	7
	118823811	121019333	2.20	81
	129082098	129135482	0.05	3
	129180275	130871839	1.69	54
5	64278949	64500585	0.22	9
	77860263	80009588	2.15	72
	96525916	98941102	2.42	50
8	135852700	136018155	0.17	4
14	1953336	3036987	1.08	35
	46176964	48062822	1.89	60
15	23234745	27699847	4.47	137
	28586239	30855591	2.27	68
	126097384	126642052	0.54	17
18	3021252	3772854	0.75	29
	4207793	4353165	0.15	4
		Total	**29.94**	961

NL-B				
**SSC**	**start**	**end**	**MB**	**NSNP**
1	61286411	63012075	1.73	47
4	57618121	57886604	0.27	8
	96094167	96243742	0.15	8
	97190659	97485594	0.29	14
	99623881	100056015	0.43	16
	102060871	103826709	1.77	68
	114814756	116278694	1.46	36
6	129636266	131919402	2.28	64
8	21169328	21827236	0.66	17
	22797483	24709455	1.91	33
	133062402	135665981	2.60	87
	135852700	136018155	0.17	4
10	61554922	61604324	0.05	3
	62611588	63274310	0.66	32
11	3154821	4191013	1.04	25
	39449593	39578555	0.13	7
13	3608413	3850825	0.24	7
14	8461080	9394510	0.93	26
	10546428	10593029	0.05	3
	46176964	47999414	1.82	58
	124728188	125116457	0.39	16
15	17425392	18252281	0.83	24
	123632778	126013489	2.38	72
	126097384	126642052	0.54	17
		Total	**22.79**	692

Table S3. Genes and QTLs mapped in the five ROH islands conserved between NL-A and NL-B pigs.

ROH location	Gene acronym or QTL ID	Gene name	Gene/QTL position
SSC1 61286411-63012075	MANEA	Mannosidase Endo-Alpha	62887689-62945586
SSC4 96094167-96243742	S100A5	S100 Calcium Binding Protein A5	96094167-96096870
	S100A6	S100 Calcium Binding Protein A6	96098264-96099686
	S100A7	S100 Calcium Binding Protein A7	96176718-96178694
	S100A8	S100 Calcium Binding Protein A8	96209670-96210603
	S100A12	S100 Calcium Binding Protein A12	96225691-96227030
	S100A9	S100 Calcium Binding Protein A9	96236114-96238989
	PPGRP-S	Porcine Peptidoglycan Recognition Protein 1	96243174-96247517
SSC8 135852700-136018155	HNRNPD	Heterogeneous Nuclear Ribonucleoprotein D	135852700-135862633
	RASGEF1B	RasGEF Domain Family Member 1B	135896022-136018155
SSC14 46176964-47999414	ZNRF3	Zinc And Ring Finger 3	46176964-46241327
	KREMEN1	Kringle Containing Transmembrane Protein 1	46254248-46323355
	EMID1	EMI Domain Containing 1	46358013-46402078
	RHBDD3	Rhomboid Domain Containing 3	46403146-46411217
	EWSR1	EWS RNA Binding Protein 1	46408636-46439357
	GAS2L1	Growth Arrest Specific 2 Like 1	46449767-46455278
	RASL10A	RAS Like Family 10 Member A	46455441-46457977
	AP1B1	Adaptor Related Protein Complex 1 Subunit Beta 1	46469462-46520175
	NEFH	Neurofilament Heavy Chain	46559698-46568686
	THOC5	THO Complex Subunit 5	46578973-46624237
	NIPSNAP1	Nipsnap Homolog 1	46624368-46641453
	NF2	NF2, Moesin-Ezrin-Radixin Like (MERLIN) Tumor Suppressor	46653869-46726421
	CABP7	Calcium Binding Protein 7	46747654-46759312
	ZMAT5	Zinc Finger Matrin-Type 5	46758436-46783055
	UQCR10	Ubiquinol-Cytochrome C Reductase, Complex III Subunit X	46783340-46786140
	ASCC2	Activating Signal Cointegrator 1 Complex Subunit 2	46797307-46842479
	MTMR3	Myotubularin Related Protein 3	46874977-47022581
	QTL #64660		46888572
	QTL #64771		47005710
	LIF	LIF Interleukin 6 Family Cytokine	47221540-47239513
	OSM	Oncostatin M	47242767-47246836
	CASTOR1	Cytosolic Arginine Sensor For MTORC1 Subunit 1	47261905-47266729
	TBC1D10A	TBC1 Domain Family Member 10A	47269087-47301540
	SF3A1	Splicing Factor 3a Subunit 1	47312460-47335448
	CCDC157	Coiled-Coil Domain Containing 157	47335500-47351171
	RNF215	Ring Finger Protein 215	47351296-47357191
	SEC14L2	SEC14 Like Lipid Binding 2	47369663-47391335
	MTFP1	Mitochondrial Fission Process 1	47392578-47396115
	GAL3ST1	Galactose-3-O-Sulfotransferase 1	47455089-47476459
	PES1	Pescadillo Ribosomal Biogenesis Factor 1	47478378-47495983
	TCN2	Transcobalamin 2	47496245-47515966
	SLC35E4	Solute Carrier Family 35 Member E4	47518602-47550157
	DUSP18	Dual Specificity Phosphatase 18	47534152-47550953
	OSBP2	Oxysterol Binding Protein 2	47558088-47737994
	MORC2	MORC Family CW-Type Zinc Finger 2	47755264-47800432
	QTL #126628		47826895
	SMTN	Smoothelin	47879317-47902700
	QTL #173177		47895001
	SELENOM	Selenoprotein M	47902862-47905350
	INPP5J	Inositol Polyphosphate-5-Phosphatase J	47912921-47922632
	PLA2G3	Phospholipase A2 Group III	47923005-47928762
	RNF185	Ring Finger Protein 185	47946370-47979702
	QTL #37844		47971494
	LIMK2	LIM Domain Kinase 2	47983984-48039631
	QTL #22109		47999414
SSC15 126097384-126642052	FAM124B	Family With Sequence Similarity 124 Member B	126112445-126127658
	CUL3	Cullin 3	126191605-126287148
	DOCK10	Dedicator Of Cytokinesis 10	126435577-126642052

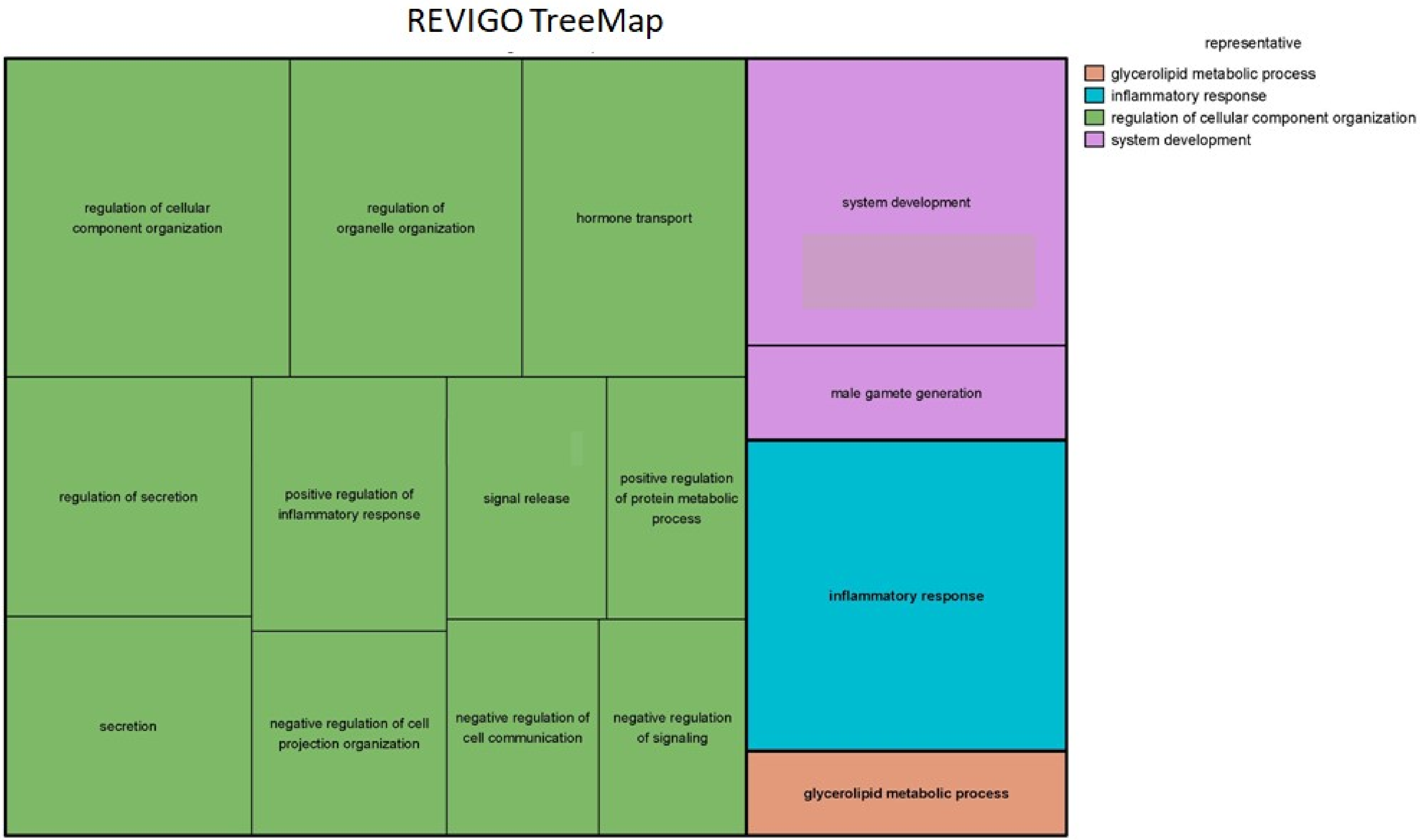

Figure S3. "Revigo Treemap" of Gene Ontology terms for genes located in the five ROH islands conserved between NL-A and NL-B pigs .

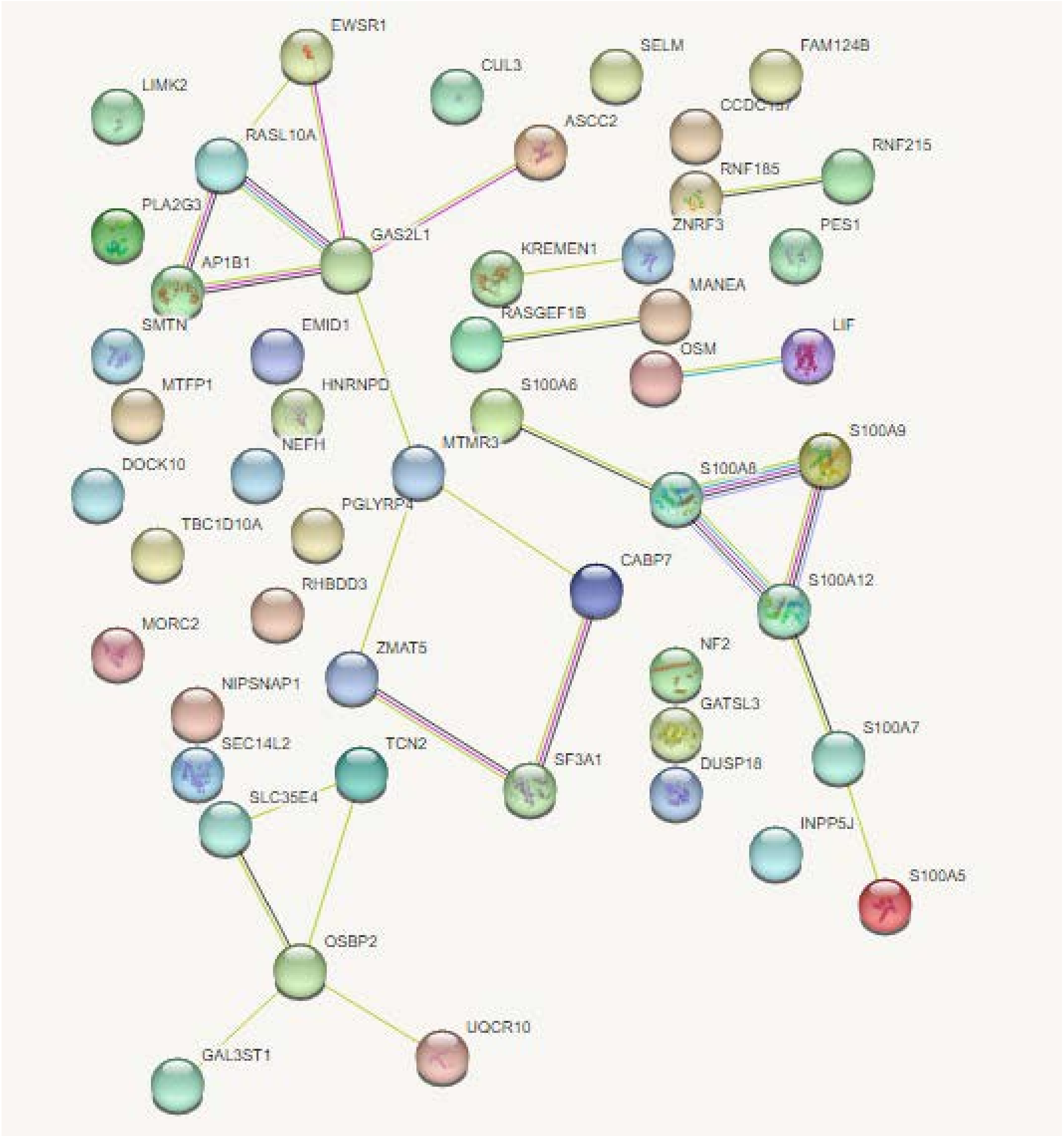

Figure S4. Network of protein–protein interaction (PPI) analysis carried out on genes located in the five ROH islands conserved between NL-A and NL-B pigs (nodes represent proteins).
